# Implementation of the nursing process in a health area: models and assessment structures used

**DOI:** 10.1590/0104-1169.3612.2479

**Published:** 2014

**Authors:** Joseba Xabier Huitzi-Egilegor, Maria Isabel Elorza-Puyadena, Jose Maria Urkia-Etxabe, Carmen Asurabarrena-Iraola

**Affiliations:** 1 PhD, Full Professor, Departamento de Enfermería II, Universidad del País Vasco, Donostia - San Sebastián, Spain; 2 PhD, Full Professor, Departamento de Física Teórica e Historia de la Ciencia, Universidad del País Vasco, Donostia - San Sebastián, Spain; 3 PhD, Professor Colaborador, Departamento de Enfermería II, Universidad del País Vasco, Donostia - San Sebastián, Spain

**Keywords:** Models, Nursing, Nursing Theory, Nursing Process, Nursing Assessment, History of Nursing, Nursing Methodology Research

## Abstract

**OBJECTIVE:**

to analyze what nursing models and nursing assessment structures have been used in the implementation of the nursing process at the public and private centers in the health area Gipuzkoa (Basque Country).

**METHOD:**

a retrospective study was undertaken, based on the analysis of the nursing records used at the 158 centers studied.

**RESULTS:**

the Henderson model, Carpenito's bifocal structure, Gordon's assessment structure and the Resident Assessment Instrument Nursing Home 2.0 have been used as nursing models and assessment structures to implement the nursing process. At some centers, the selected model or assessment structure has varied over time.

**CONCLUSION:**

Henderson's model has been the most used to implement the nursing process. Furthermore, the trend is observed to complement or replace Henderson's model by nursing assessment structures.

## Introduction

The nursing process is the work method used in nursing and permits, through research, logical analysis and analytic reasoning, the development and application of technical, interpersonal or relational and communication care. It involves five steps: assessment, diagnosis, planning, execution and, finally, evaluation^(^ 1 ^)^.

Its creation dates back to the 1950-1960's in the United States of America and Canada^(^ 2 ^)^. In the initial years, it is mainly discussed and developed in teaching. In the 1970's, it is extended to clinical practice (professional practice) in those countries and continues developing. The establishment of the nursing diagnoses and NIC-NOC terminologies are steps that stand out in this development. Nowadays, the nursing process is used at health centers all over the world^(^ 3 ^-^ 4 ^)^.

In the same decades as the creation and the implementation of the nursing process, models and theories are established that serve as a guide for professional nursing practice^(^ 5 ^)^. While the nursing process is a method to organize professional practice, the nursing models and theories are a framework to understand and give meaning to this professional practice, which guarantees a strict practice based on personal experience and on the scientific and philosophical premises each theorist contributes. Therefore, one should depart from general philosophical and scientific elements that will transform into concrete and unique elements, which will serve to define the empirical and operative elements of each conceptual model. In that sense, the nursing guides or models used condition the practice, which rests on the theoretical foundations each model contains and which the nurses identify with, accepting that the clients and their own reality are conditioned by the underlying theoretical base^(^ 6 ^)^.

In general, one might say that the nursing models and theories help to identify the objectives and practice means each person is provided with^(^ 7 ^)^. To give an example, when adopting the Henderson model, the nurses accept the proposal to satisfy the patients' needs and aim to generate the independence of each person. If they prefer Orem's model, they will aim to maximize the clients' self-care skills^(^ 6 ^)^.

Later, without reaching the point of being models or theories, different nursing assessment structures are published, including Carpenito's bifocal structure^(^ 8 ^)^ and Gordon's conceptual structure^(^ 9 ^)^. The bifocal structure Carpenito introduced in 1983 is a model in which the clinical situations are identified in which the nurse intervenes, making reference to independent nursing work and interdependent or collaborative work. Gordon's conceptual structure serves as a general reference framework to assess each person through the proposed Functional Health Patterns. After obtaining Gordon's permission and after being modified by NANDA International's taxonomy committee, the NANDA accepted this structure in April 1998^(^ 10 ^)^.

Few studies^(11-12) ^have been found in the Spanish context that contribute with numerical data about the nursing models and structures used in the application of the nursing method. These studies are not monographs about the theme and do not indicate concrete start dates.

The objective in this research is to study what nursing models and structures have been used to implement the nursing process at the public and private centers in the health area Gipuzkoa (Basque Country). The results will reveal the evolution in the nursing work mode in Gipuzkoa and the past and current presence of the nursing models and structures in the nursing method.

## Method

A retrospective study was undertaken about the nursing records used at the public and private centers in the health area Gipuzkoa, one of the three health areas in the Basque Country.

In total, 158 centers were studied: 137 public (all public centers) and 21 private (all centers with a staff of 10 or more nursing professionals). At these 158 centers, 2667 nursing professionals work (2103 at the public centers and 564 at the private centers), which correspond to 90% of all professionals working in clinical practice in Gipuzkoa.

The nursing records were obtained or visualized after getting authorization from the management of the involved centers, and after interviewing the professionals responsible for these records. The anonymity of the centers was preserved in the publication of the data.

The Research Ethics Committee at the Universidad del País Vasco - Euskal Herriko Unibertsitatea (UPV-EHU) started its activities in 2010. The data for this study were collected between January and December 2009, which is why the assessment of this committee was not possible.

The following data were collected for each center:

-The number of nursing professionals working at the center.
-The date of the oldest nursing assessment records existing at the center. The existence of records corresponding to the nursing assessment phase was the criterion followed to consider that the nursing process is applied, as the rest of the nursing process phases rest on the first^(^ 1 ^,^ 13 ^)^.
-The shape of the nursing assessment records used at the center over time. To determine what nursing model and/or what assessment structure were used, it was verified that the shape of the records corresponds to the guidelines that mark one or another model or structure. To give an example, it was considered that Henderson's model was used if the assessment records address at least 75% of the basic needs presented in that model^(^ 14 ^)^. In another example, it was considered that Gordon's assessment structure was used if at least 75% of the functional patterns this structure rests on were assessed.

Quantitative analysis was applied to the data, using descriptive statistics.

## Results

In Table 1, the centers studied are shown, as well as the number of nursing professionals, the year the application of the nursing process (NP) started, the nursing model or assessment structure used in the application of the nursing process and the year this nursing model or assessment structure started to be used. When the cell is empty, this means that the above are not used at the center. The number of nursing professionals working at each center reveals its size. 

**Table 1:** - Centers studied, number of nursing professionals working there, start year of application of the nursing process (NP), nursing model or assessment structured used at the time of the research in the application of the nursing process and start year of this nursing model or assessment structure. Gipuzkoa, Basque Country, Spain, 2009

Health system and area			No. of centers	No. of nursing professionals	Start year of NP	Model or structure used today	Start year of this model or structure
Public health system			137	2103			
	Primary care		116	514	2004	Henderson	2004
	Specialized care		5	1523	1990	Bifocal*	1994
		Hospital No. 1	1	1072	1990	Bifocal*	1994
		Hospital No. 2	1	110	1990	Bifocal*	1994
		Hospital No. 3	1	111	1990	Bifocal*	1994
		Hospital No. 4	1	143	1990	Bifocal*	1994
		Hospital No. 5	1	87	1990	Bifocal*	1994
	Mental health		15	54			
		Psychiatry service Hospital No. 1	1	22	1990	Bifocal*	1994
		Outpatient care centers	14	32	2007	Gordon	2007
	Geriatrics		1	12	1990	Henderson	1996
Private health system			21	564			
	Specialized care		6	410			
		Hospital No. 1	1	35	-	-	-
		Hospital No. 2	1	85	-	-	-
		Hospital No. 3	1	40	2003	Henderson	2003
		Hospital No. 4	1	50	1993	-	-
		Hospital No. 5	1	165	-	-	-
		Hospital No. 6	1	35	2004	Gordon	2004
	Mental health		4	69			
		Hospital No. 1	1	25	1992	Henderson	1992
		Hospital No. 2	1	22	1998	Gordon^†^	2005
		Hospital No. 3	1	12	2005	Gordon	2005
		Hospital No. 4	1	10	2001	-	-
	Geriatrics		11	85			
		Geriatric center No. 1	8	55	1982	Henderson	1982
		Geriatric center No. 2	1	10	1999	RAI-NH2.0	1999
		Geriatric center No. 3	1	10	2002	-	-
		Geriatric center No. 4	1	10	1994	-	-
							

* At these centers, between 1990 and 1994, Henderson's nursing model was used. In 1994, Carpenito's bifocal structure started to be used.

† At this center, between 1998 and 2005, Henderson's nursing model was used. In 2005, this model stopped being used and Gordon's assessment structure started to be used.

According to the figures in Table 1, besides some exception, the use of the nursing process starts in 1990 and is generalized in the first decade of the 21^st^ century. Nowadays, at 155 (98%) out of the 158 centers studied, the nursing process is applied. It is applied at all public centers and 18 out of 21 private centers. The details of this start are available for one public center and 18 out of 21 private centers. These details are available in a previously published retrospective study^(^ 15 ^)^, and the details of the current use (the use of the nursing diagnoses, the NIC-NOC terminologies and the standardized care plans) in an also published cross-sectional study^(^ 12 ^)^.

At 127 (82%) out of the 155 centers that apply the nursing process, Henderson's model is used; at six (4%) Carpenito's bifocal structure is used; at 17 (11%) Gordon's conceptual structure and, at one, the RAI-NH 2.0 structure (*Resident Assessment Instrument Nursing Home 2.0). *At the remaining four centers, the nursing process is applied without the support of any model or assessment structure, with records being elaborated according to the particular criterion of the nursing professionals at each center.

Carpenito's bifocal structure, also known as the *bifocal model of clinical practice*, is an assessment structure that proposes the use of a nursing model to recollect that data that permit obtaining the client's nursing diagnoses and the use of the body system assessment structure to collect the data that permit obtaining the interdependent problems or the client's cooperation. At the public specialized care centers and the psychiatry service of hospital No. 1 studied, Carpenito's bifocal structure has been implemented as follows: Henderson's model has been used to collect the data that permit reaching the client's nursing diagnoses, and the body system assessment structure has been used to collect the data that permit reaching the client's interdependent problems. Therefore, keeping in mind that the centers that use Carpenito's bifocal structure are applying Henderson's model, when adding up the 127 centers where Henderson's model is used and the six centers where Carpenito's bifocal structure is applied, today, among the 155 centers that apply the nursing process, at 133 (86%), Henderson's model is used.

In terms of the number of nursing professionals, at the centers where the nursing process is applied with the sole use of Henderson's model, 578 professionals are active (24% of all professionals who work at the 155 centers where the nursing process is applied); at the centers where Carpenito's bifocal structure is used, 1545 (65%); at the centers where Gordon's conceptual structure is used, 101 (4%) and, at the center where the RAI-NH 2.0 structure is used, 10 (0.4%). When adding up the 578 nursing professionals who work at the centers where the nursing process is applied using Henderson's model and the 1545 professionals who work at the centers where the nursing process is applied using Carpenito's bifocal structure, today, 2123 (89%) of the professionals work at centers that use Henderson's model.

Concerning the start year of the model or structure, it should be highlighted that, at some centers, the selected model or structure has varied over time: at the public specialized care center or at the psychiatry service of public hospital No. 1, in 1990-1994, Henderson's nursing model was used and, as from 1994, Carpenito's bifocal structure; at private mental health hospital No. 2, in 1998-2005, Henderson's nursing model was used and, as from 2005, Gordon's conceptual structure.

When considering the activity area, it is observed that Henderson's nursing model is used in all areas; the bifocal structure is mainly used in the specialized care area; Gordon's conceptual structure is mainly used in the mental health area and the RAI-NH 2.0 structure in geriatrics.

## Discussion

The results show that, in Gipuzkoa, Henderson's model has been the most used in the implementation of the nursing process. That is in accordance with the authors who refer that Henderson's model is one of the most well-known and globally used models^(^ 5 ^,^ 16 ^)^.

The usage figures obtained in this study, superior to 80% in the case of Henderson's model, is higher than the figures obtained in other similar studies. In a study undertaken in Spain, it is affirmed that, in primary care, 35% of the centers use Henderson's model, which is the only one used^(^ 11 ^)^. In a study undertaken in Canada, it was found that only 25.5% of the centers use a nursing model to apply the nursing process^(^ 17 ^)^. The study does not specify what nursing models are used. In another study developed in Poland, it is concluded that, in that country's clinical practice, the ideas and theories of Nightingale, Orem and Henderson are used, but the study does not quantify this use per centers^(^ 18 ^)^.

In general, in Gipuzkoa, the nursing process is applied under the influence of a nursing model or theory. Exceptions are the centers that apply the nursing process using Gordon's conceptual structure, the RAI-NH 2.0^(^ 19 ^)^ structure or with records made in line with the center professionals' private criterion. It should be kept in mind that Gordon's conceptual structure and the RAI-NH 2.0 structure are not actually nursing models, but nursing assessment structures.

In view of the above, those centers should be focused on which, over time, have varied their choice of the model or assessment structure. These variations have affected 1567 nursing professionals (66% of all professionals who work at the 155 centers who apply the nursing process), and are related either to replacing Henderson's model by an assessment structure or to complementing Henderson's model with Carpenito's bifocal structure. It seems that, at these centers, Henderson's model alone was not sufficient for the correct assessment of the patient-client, and that something more or something different was required. Therefore, in Gipuzkoa, the trend is observed to complement or replace Henderson's model by nursing assessment structures.

This trend appears to confirm those authors who alert to the problems that can emerge if the selected model is not used correctly or who indicate difficulties when combining the nursing model with the incorporation of novelties (the nursing diagnoses, the NIC-NOC terminologies) related to the nursing process^(^ 20 ^-^ 21 ^)^.

Concerning the activity area, as mentioned, Gordon's conceptual structure is mainly used in the mental sector. This can be due to the fact that the school in the Basque Country that offers the programs to obtain the degree of mental health nursing specialist has selected this structure.

As regards the geriatric area, it should be observed that, at the public as well as the private centers, in the nursing assessment records, a paragraph was found in which the patients' ability to accomplish the activities of daily living is assessed. This term "activities of daily living" reminds of the term "activities of living" in the nursing model by Roper-Logan-Tierney^(^ 22 ^)^ but, in the records studied, the 12 activities of living this model proposes are not assessed, but only those activities that propose the indices used to measure the autonomy in the accomplishment of the activities of daily living (Katz, Barthel and Lawton indices). That is the reason why it was not considered that this nursing model is applied.

Finally, it is noteworthy that, at almost all public centers in the same area, the selected assessment model or structure coincides and is simultaneous over time. At the private centers, on the other hand, there are differences in the choice of the model or assessment structure as well as in the start date. That is so because, at the public centers, the management of each area, which drives the changes, is unified. The management at the private centers, on the other hand, is independent, without any connections among them.

## Conclusions

These study results show that, in Gipuzkoa, Henderson's model has been the most used in the implementation of the nursing process.

Over time, a trend is observed to complement or replace Henderson's model by nursing assessment structures.

Finally, this study reveals that, at the public centers in the same area, the nursing model or assessment structure almost always coincides, which is not the case at the private centers.

## References

[1] Alfaro-Lefevre R (2014). Applying nursing process: the foundation for clinical
reasoning.

[2] De la Cuesta C (1983). The Nursing Process: from development to
implementation. J Adv Nurs..

[3] Müller-Staub M, Lavin MA, Needham I, Van Achterberg T (2006). Nursing diagnoses, interventions and outcomes -
application and impact on nursing practice: systematic review. J Adv Nurs..

[4] Lunney M (2003). Critical thinking and accuracy of nurses
diagnosis. Int J Nurs Terminol Classif..

[5] Raile M, Marriner A (2010). Nursing theorists and their work.

[6] Durán de Villalobos M (2009). Marco epistemológico de la enfermería. Aquichan. [Internet].

[7] Meleis A (2012). Theoretical nursing development and progress.

[8] Carpenito LJ (2013). Nursing diagnosis: Application to clinical practice.

[9] Gordon M (2010). Manual of nursing diagnosis.

[10] NANDA-International (2013). Diagnósticos enfermeros.

[11] Izquierdo JM, Pérez MB, Ramírez FJ, Serrano I, Torres MD, Conde G (2002). Implantación del proceso enfermero. Rol Enferm..

[12] Huitzi-Egilegor JX, Elorza-Puyadena MI, Urkia-Etxabe JM, Zubero-Linaza J, Zupiria-Gorostidi X (2012). Use of the nursing process at public and private centers
in a health area. Rev. Latino-Am. Enfermagem..

[13] Giménez AM, Serrano P (2009). Imprecisiones del proceso diagnóstico
enfermero. Metas Enferm. dic 2008/ene.

[14] De Henderson VA (1991). The nature of nursing: Reflections after 25 years.

[15] Huitzi-Egilegor JX, Elorza-Puyadena MI, Urkia-Etxabe JM, Esnaola-Herrero MV, Asurabarrena-Iraola C (2013). Retrospective study of the implementation of the nursing
process in a health area. Rev. Latino-Am. Enfermagem..

[16] Luis MT, Navarro MV, Fernández C (2005). De la teoría a la práctica: el pensamiento de Virginia
Henderson en el Siglo XXI.

[17] Simpson J, Taylor D (2002). Reality check. Do conceptual models of nursing work
today?. Can Nurse..

[18] Zarzycka D, Dobrowolska B, Slusarska B, Wronska I, Cuber T, Pajnkihar M (2013). Theoretical foundations of nursing practice in
Poland. Nurs Sci Q..

[19] Morris JN, Hawes C, Fries BE, Phillips CD, Mor V, Katz S (1990). Designing the national resident assessment instrument
for nursing homes. Gerontologist..

[20] Kalisch BJ, Landstrom GL, Hinshaw AS (2009). Missed nursing care: a concept analysis. J Adv Nurs..

[21] Reyes J, Jara P, Merino JM (2007). Adherencia de las enfermeras/os a utilizar un modelo
teórico como base de la valoración de enfermería. Cienc Enferm. [Internet].

[22] Roper N, Logan WW, Tierney AJ (2009). The Roper-Logan-Tierney model of nursing: based on activities
of living.

